# Protocol for improving diffraction quality of leucyl-tRNA synthetase 1 with methylation and post-crystallization soaking and cooling in cryoprotectants

**DOI:** 10.1016/j.xpro.2021.100642

**Published:** 2021-07-02

**Authors:** Sulhee Kim, Ina Yoon, Sunghoon Kim, Kwang Yeon Hwang

**Affiliations:** 1 Division of Biotechnology, College of Life Sciences and Biotechnology, Korea University, Seoul 02841, Republic of Korea; 2 Institute of Life Science and Natural Resources, Korea University, Seoul 02841, Republic of Korea; 3 Medicinal Bioconvergence Research Center, Institute for Artificial Intelligence and Biomedical Research, College of Pharmacy & College of Medicine, Gangnam Severance Hospital, Yonsei University, Incheon 21983, Republic of Korea

**Keywords:** Protein Biochemistry, Structural Biology, X-ray Crystallography

## Abstract

Leucyl-tRNA synthetase 1 (LARS1) synthesizes Leu-tRNA^Leu^ for protein synthesis and plays an important role in mTORC1 activation by sensing intracellular leucine concentrations. Here, we describe a protocol for the purification, reductive methylation, binding affinity measurement by microscale thermophoresis, T_*i*_ value measurement by Tycho, and post-crystallization soaking and cooling in cryoprotectants to improve crystallization of LARS1. Collectively, this allowed us to build the RagD binding domain, which was shown to be a dynamic region of LARS1 refractory to crystallization.

For complete details on the use and execution of this protocol, please refer to Kim et al. (2021).

## Highlights

• Efficient reductive methylation of LARS1 for improvement of crystal resolution
• Optimized post-crystallization soaking and cooling in cryoprotectants with crystals
• Biochemical binding affinity measurement of LARS1 by microscale thermophoresis

## Graphical abstract

**Figure:**
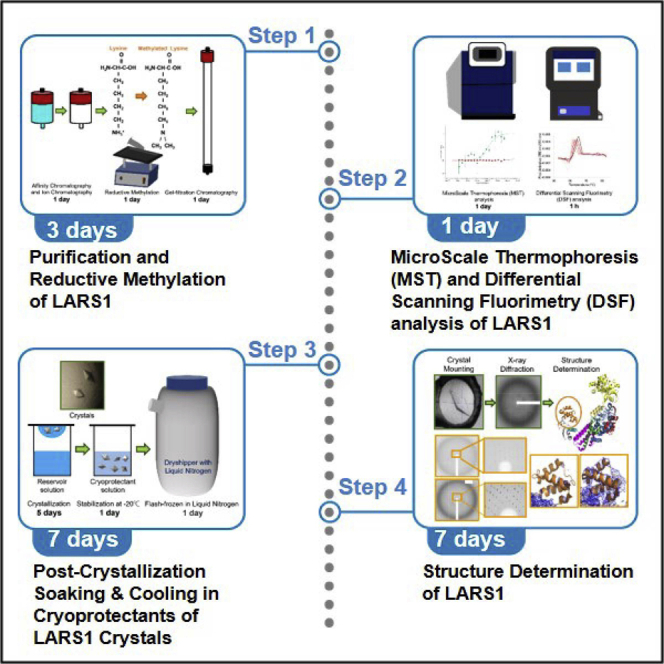

## Before you begin

**Figure 1:**
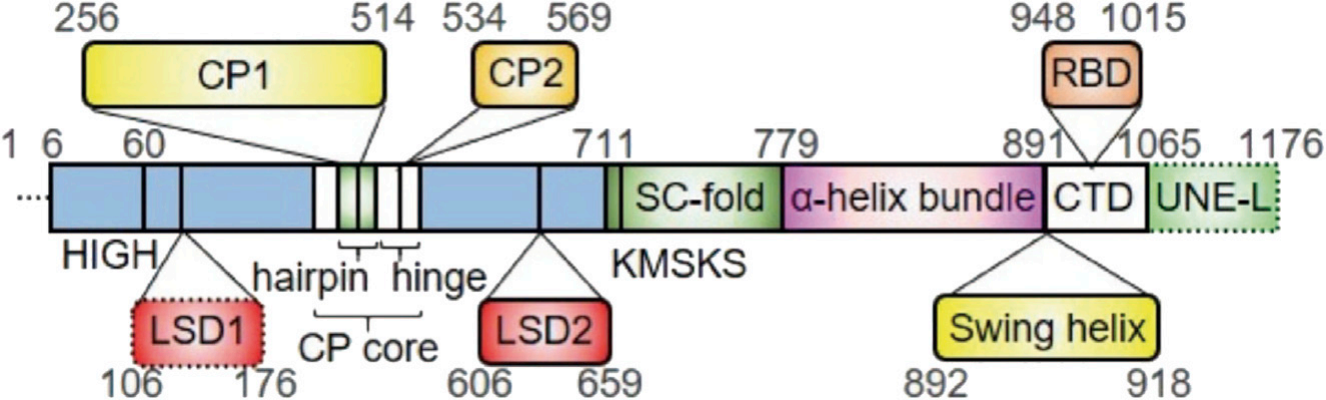
Overall Domain arrangement of LARS1 CD, catalytic domain; LSD, leucine-specific domain; CP, connective polypeptide; SC, stem-contact; CTD, C-terminal domain; RBD, RagD-binding domain; UNE-L, unnamed domain of LARS. Figure reprinted with permission from Kim et al. (2021).

LARS1 protein is composed of 1,176 amino acid residues, and contains a catalytic domain, an editing domain, a dynamic C-terminal domain composed of a RagD-binding domain (RBD), LVβ, and UNE-L domains. LARS1 is a multidomain and flexible protein (Figure 1); therefore, the X-ray structure of LARS1 has not been solved for a long time. Recently, we were able to obtain crystals of LARS1 complexed with leucine, Leu-AMS, and ATP, respectively, and improved crystal behavior by reductive methylation of lysine and post-crystallization soaking & cooling in cryoprotectants at −20°C. We also measured the binding affinity of leucine for LARS1 using MicroScale Thermophoresis (MST) and checked T_*i*_ values using differential scanning fluorimetry (DSF). In this protocol, we describe a procedure for the purification and the reductive methylation (Walter et al., 2006; Kobayashi et al., 1999)) of LARS1, and measurements by MST and DSF. We present the crystallization and structure determination of LARS1 with leucine, Leu-AMS, and ATP. To begin, we needed to generate the expression plasmid by performing DNA cloning and transformation.

### Plasmid transformation of *E. coli* Rosetta (DE3)

**Timing: 1 day**

In this step, Rosetta (DE3) competent *E. coli* cells were transformed with a pQE80L plasmid vector containing cDNA encoding human *LARS1* (amino acids–1-1176) at the *BamH*I/*Hind*III restriction sites.

1. Remove Rosetta (DE3) competent cells from −80°C freezer and thaw on ice.
2. Add 1 μL recombinant plasmid (∼100 ng/μL) to 50 μL of competent cells in a microcentrifuge tube.
3. Incubate on ice for 30 min.
4. Heat shock at 42°C for 2 min.
5. Incubate on ice for 10 min.
6. Add 100 μL LB media to the tube.
7. Incubate the tube at 37°C in an incubator with mechanical shaking at 180 rpm for 1 h.
8. Spread cells onto an LB agar plate containing 100 μg/mL ampicillin and 100 μg/mL chloramphenicol.
9. Incubate the plate at 37°C for 16–18 h.

### Expression of LARS1 in *E. coli*

**Timing: 2 days**

10. Pick a single colony with a sterile tip from the agar plate and inoculate into 100 mL LB broth containing 100 μg/mL ampicillin and 100 μg/mL chloramphenicol in a 500 mL baffled flask.
11. Incubate the culture at 37°C with shaking at 180 rpm for 16–18 h until optical density at 600 nm (OD600) reaches approximately 3.0.
12. Transfer 10 mL of the culture into 1 L of LB broth containing 100 μg/mL ampicillin and 100 μg/mL chloramphenicol in a 2 L baffled flask.
13. Incubate the culture for 2 h at 37°C with shaking at 180 rpm until the OD_600_ reaches approximately 0.6–0.8.
14. Cool down the culture to ∼18°C for 4 h.
15. Induce the cell culture with 500 μL of 1 M isopropyl β-D-1-thiogalactopyranoside (IPTG) at a final concentration of 0.5 mM.
16. Incubate the culture for 18 h at 18°C with shaking at 180 rpm.
17. Harvest cells by centrifugation at 4,500 × *g* for 20 min at 4°C.
18. Remove the supernatant and transfer the pellet to a 50 mL centrifuge tube.
19. Store at −20°C for subsequent purification.

## Key resources table

**Table:** 

REAGENT or RESOURCE	SOURCE	IDENTIFIER
**Bacterial and viral strains**		

*E. coli*: Rosetta (DE3)	Novagen	70954

**Chemicals, peptides, and recombinant proteins**		

Leu-AMS	(Kim et al., 2021)	N/A
LB broth	Merck Millipore	Cat# 1102855000
Agar	Duchefa	Cat# M1002.1000
Ampicillin	Duchefa	Cat# A0104.0025
Chloramphenicol	Sigma-Aldrich	Cat# C0378-100G
IPTG	Duchefa	Cat# 11401.0025
L-Leucine	Sigma-Aldrich	Cat# L8000-100G
β-Mercaptoethanol	Sigma-Aldrich	Cat# M3148-250ML
Tween-20	Sigma-Aldrich	Cat# P1379-1L
Imidazole	Bio Basic	Cat# IB0277-500G
Hydrochloric acid	Samchun	Cat# H0255
Sodium hydroxide	Samchun	Cat# S0610
DTT	Duchefa	Cat# D1309.0025
TRIS-HCl	Duchefa	Cat# T1501.5000
HEPES	Merck Millipore	Cat# 391338-1KGCN
Sodium chloride	Duchefa	Cat# S0520.5000
Magnesium chloride	Duchefa	Cat# M0533.1000
Formaldehyde	Sigma-Aldrich	Cat# F8775-25ML
Borane dimethylamine complex (DMAB)	Sigma-Aldrich	Cat# 180238-25G
ATP	Sigma-Aldrich	Cat# A2383-25G
Bis-tris (pH6.5)	Hampton Research	Cat# HR2-783
Ammonium sulfate	Hampton Research	Cat# HR2-541
HEPES	Hampton Research	Cat# HR2-585
PEG3350	Hampton Research	Cat# HR2-527
Sodium hydroxide	Hampton Research	Cat# HR2-583
Glycerol	Hampton Research	Cat# HR2-623
BSA	Sigma-Aldrich	Cat# A1933
Index	Hampton Research	Cat# HR2-144
His-Tag Labeling Kit RED-tris-NTA 2^nd^ Generation	NanoTemper	Cat# MO-L018

**Deposited data**		

Structure of human LARS1-Leu^syn^	Protein Data Bank (PDB)	PDB: 6KQY
Structure of human LARS1-ATP^syn^	Protein Data Bank (PDB)	PDB: 6KID
Structure of human LARS1-Leu-AMS^syn^	Protein Data Bank (PDB)	PDB: 6KIE
Structure of human LARS1^methyl^-Leu-AMS^syn^	Protein Data Bank (PDB)	PDB: 6KR7

**Recombinant DNA**		

Plasmid: pQE-80L-His-LARS1	(Han et al., 2012)	N/A

**Software and algorithms**		

HKL2000	(Otwinowski and Minor, 1997)	http://www.hkl-xray.com/
AutoSol	(Adams et al., 2002)	https://www.phenix-online.org/
PHENIX	(Liebschner et al., 2019)	https://www.phenix-online.org/
Phaser	(Adams et al., 2002)	https://www.phenix-online.org/
COOT	(Emsley and Cowtan, 2004)	https://www2.mrc-lmb.cam.ac.uk/personal/pemsley/coot/
PyMOL	(Rigsby and Parker, 2016)	https://www.pymol.org/2/
Monolith NT.115	MO.Affinity Analysis v2.3	NanoTemper Technologies, Munich, Germany
Tycho NT.6	Tycho Analysis	NanoTemper Technologies, Munich, Germany

**Other**		

HisTrap HP	Cytiva (GE Healthcare)	Cat# 17-5248-02
HiTrap Q FF	Cytiva (GE Healthcare)	Cat# 17-5156-01
HiLoad 26/600 Superdex 200 pg	Cytiva (GE Healthcare)	Cat# 28-9898-36
Amicon Ultra-15 Centrifugal Filter; 30kDa cutoff	Merck Millipore, GE	Cat# UFC903096
Millex-HV Syringe Filter Unit, 0.45 μm	Merck Millipore, GE	Cat# SLHV004SL
MRC 2 Well Crystallization Plate	Hampton Research	Cat# HR3-083
VDX^TM^ Plate with sealant	Hampton Research	Cat# HR3-171
9-Well glass plate	Hampton Research	Cat# HR3-134
22 mm × 0.22 mm Siliconized square cover slides	Hampton Research	Cat# HR3-217
Monolith NT.115 Capillaires	NanoTemper	Cat# MO-K022
Tycho NT.6 Capillaries	NanoTemper	Cat# TY-C001
AKTA Prime Plus FPLC System	Cytiva (GE Healthcare)	N/A
AKTA Purifier 100 FPLC System	Cytiva (GE Healthcare)	N/A

## Materials and equipment

### Lysis buffer A

Lysis buffer A contains 20 mM Tris-HCl, pH 8.0, 150 mM NaCl, 5 mM MgCl_2_, 5 mM β-mercaptoethanol, and 0.1% Tween-20. Prepare 1 L of lysis buffer A:

**Table:** 

Reagents	Amount	Final concentration
Tris-base	2.42 g	20 mM
NaCl	8.77 g	150 mM
MgCl_2_	1.02 g	5 mM
β-mercaptoethanol	350 μL	5 mM
Tween-20	1 mL	0.1%

The buffer can be stored at 4°C for 1 day.

**CRITICAL:** β-mercaptoethanol should be added to the buffer immediately before use.

### Histrap binding buffer A

Histrap binding buffer A contains 20 mM Tris-HCl, pH 8.0, 150 mM NaCl, 5 mM MgCl_2_, 5 mM β-mercaptoethanol, and 0.1% Tween-20. Prepare 1 L of Histrap binding buffer A:

**Table:** 

Reagents	Amount	Final concentration
Tris-base	2.42 g	20 mM
NaCl	8.77 g	150 mM
MgCl_2_	1.02 g	5 mM
β-Mercaptoethanol	350 μL	5 mM
Tween-20	1 mL	0.1%

The buffer can be stored at 4°C for 1 day.

### Histrap wash buffer A

Histrap wash buffer A contains 20 mM Tris-HCl, pH 8.0, 150 mM NaCl, 5 mM MgCl_2_, 5 mM β-mercaptoethanol, 0.1% Tween-20, and 20 mM imidazole. Prepare 500 mL of Histrap wash buffer A:

**Table:** 

Reagents	Amount	Final concentration
Tris-base	1.21 g	20 mM
NaCl	4.38 g	150 mM
MgCl_2_	0.51 g	5 mM
β-mercaptoethanol	175 μL	5 mM
Tween-20	0.5 mL	0.1%
Imidazole	0.68 g	20 mM

The buffer can be stored at 4°C for 1 day.

### Histrap elution buffer A

Histrap elution buffer A contains 20 mM Tris-HCl, pH 8.0, 150 mM NaCl, 5 mM MgCl_2_, 5 mM β-mercaptoethanol, 0.1% Tween-20, and 500 mM imidazole. Prepare 500 mL of Histrap elution buffer A:

**Table:** 

Reagents	Amount	Final concentration
Tris-base	1.21 g	20 mM
NaCl	4.38 g	150 mM
MgCl_2_	0.51 g	5 mM
β-Mercaptoethanol	175 μL	5 mM
Tween-20	0.5 mL	0.1%
Imidazole	17.02 g	500 mM

The buffer can be stored at 4°C for 1 day.

### Q binding buffer A

Q binding buffer A contains 20 mM Tris-HCl, pH 8.0, 2 mM DTT, and 0.1% Tween-20. Prepare 1 L of Q binding buffer A:

**Table:** 

Reagents	Amount	Final concentration
Tris-base	2.42 g	20 mM
DTT	0.31 g	2 mM
Tween-20	1 mL	0.1%

The buffer can be stored at 4°C for 1 day.

### Q elution buffer A

Q elution buffer A contains 20 mM Tris-HCl, pH 8.0, 1 M NaCl, 5 mM MgCl_2_, 2 mM DTT, and 0.1% Tween-20. Prepare 500 mL of Q elution buffer A:

**Table:** 

Reagents	Amount	Final concentration
Tris-base	1.21 g	20 mM
NaCl	29.22	1 M
MgCl_2_	0.51 g	5 mM
DTT	0.15 g	2 mM
Tween-20	0.5 mL	0.1%

The buffer can be stored at 4°C for 1 day.

### Gel-filtration buffer

Gel-filtration buffer contains 20 mM Tris-HCl, pH 7.5, 150 mM NaCl, 5 mM MgCl_2_, 2 mM DTT, and 0.1% Tween-20. Prepare 1 L of gel-filtration buffer:

**Table:** 

Reagents	Amount	Final concentration
Tris-base	2.42 g	20 mM
NaCl	8.77 g	150 mM
MgCl_2_	1.02 g	5 mM
DTT	0.31 g	2 mM
Tween-20	1 mL	0.1%

The buffer can be stored at 4°C for 1 day.

### Lysis buffer B (for methylation)

Lysis buffer B contains 50 mM HEPES, pH 7.5, 150 mM NaCl, 5 mM MgCl_2_, 5 mM β-mercaptoethanol, and 0.1% Tween-20. Prepare 1 L of lysis buffer B:

**Table:** 

Reagents	Amount	Final concentration
HEPES	11.92 g	50 mM
NaCl	8.77 g	150 mM
MgCl_2_	1.02 g	5 mM
β-mercaptoethanol	350 μL	5 mM
Tween-20	1 mL	0.1%

The buffer can be stored at 4°C for 1 day.

### Histrap binding buffer B (for methylation)

Histrap binding buffer B contains 50 mM HEPES, pH 7.5, 150 mM NaCl, 5 mM MgCl_2_, 5 mM β-mercaptoethanol, and 0.1% Tween-20. Prepare 1 L of Histrap binding Buffer B:

**Table:** 

Reagents	Amount	Final concentration
HEPES	11.92 g	50 mM
NaCl	8.77 g	150 mM
MgCl_2_	1.02 g	5 mM
β-mercaptoethanol	350 μL	5 mM
Tween-20	1 mL	0.1%

The buffer can be stored at 4°C for 1 day.

### Histrap wash buffer B (for methylation)

Histrap wash buffer B contains 50 mM HEPES, pH 7.5, 150 mM NaCl, 5 mM MgCl_2_, 5 mM β-mercaptoethanol, 0.1% Tween-20, and 20 mM imidazole. Prepare 500 mL of Histrap wash buffer B:

**Table:** 

Reagents	Amount	Final concentration
HEPES	5.96 g	50 mM
NaCl	4.38 g	150 mM
MgCl_2_	0.51 g	5 mM
β-mercaptoethanol	175 μL	5 mM
Tween-20	0.5 mL	0.1%
Imidazole	0.68 g	20 mM

The buffer can be stored at 4°C for 1 day.

### Histrap elution buffer B (for methylation)

Histrap elution buffer B contains 50 mM HEPES, pH 7.5, 150 mM NaCl, 5 mM MgCl_2_, 5 mM β-mercaptoethanol, 0.1% Tween-20, and 500 mM imidazole. Prepare 500 mL of Histrap elution buffer B:

**Table:** 

Reagents	Amount	Final concentration
HEPES	5.96 g	50 mM
NaCl	4.38 g	150 mM
MgCl_2_	0.51 g	5 mM
β-mercaptoethanol	175 μL	5 mM
Tween-20	0.5 mL	0.1%
Imidazole	17.02 g	500 mM

The buffer can be stored at 4°C for 1 day.

### Q binding buffer B (for methylation)

Q binding buffer B contains 50 mM HEPES, pH 7.5, 2 mM DTT, and 0.1% Tween-20. Prepare 1 L of Q binding buffer B:

**Table:** 

Reagents	Amount	Final concentration
HEPES	11.92 g	50 mM
DTT	0.31 g	2 mM
Tween-20	1 mL	0.1%

The buffer can be stored at 4°C for 1 day.

### Q elution buffer B (for methylation)

Q elution buffer B contains 50 mM HEPES, pH 7.5, 1 M NaCl, 5 mM MgCl_2_, 2 mM DTT, and 0.1% Tween-20. Prepare 500 mL of Q elution buffer B:

**Table:** 

Reagents	Amount	Final concentration
HEPES	5.96 g	50 mM
NaCl	29.22	1 M
MgCl_2_	0.51 g	5 mM
DTT	0.15 g	2 mM
Tween-20	0.5 mL	0.1%

The buffer can be stored at 4°C for 1 day.

### Methylation solution

Dimethylamine borane complex (DMAB) (1 mL)

**Table:** 

Reagents	Amount	Final concentration
Dimethylamine borane complex (DMAB)	58.9 mg	1 M

DMAB solution can be stored at 4°C for 1 day.

**Table:** Formaldehyde (1 mL)

Reagent	Amount	Final concentration
16% formaldehyde solution	0.188 mL	1 M

Formaldehyde solution can be stored at 4°C for 2–3 months.

**Table:** Tris-HCl, pH 7.5 (1 mL)

Reagent	Amount	Final concentration
**Tris-base**	121 mg	1 M

This buffer can be stored at 4°C for 2–3 month.

**Table:** DTT (1 mL)

Reagent	Amount	Final concentration
DTT	7.7 mg	50 mM

DTT solution can be stored at −20°C for 2–3 months.

## Step-by-step method details

### Purification of leucyl-tRNA synthetase 1 (LARS 1)

**Timing: 2 days**

In this step, the frozen cells obtained from the bacterial expression of LARS1 are purified by affinity chromatography, ion chromatography, and gel filtration chromatography.

1. Preparation of cell lysate.
  a. Thaw the frozen cells on ice.
  b. Resuspend the cells in 50 mL lysis Buffer A per L cell culture.
  c. Transfer the resuspended cells into a beaker placed on ice.
  d. Sonicate cells for 6 min at 60 amplitudes with intervals of 2 s on and 6 s off.
    **CRITICAL:** It is critical to maintain cells at a low temperature. Keep the cells on ice.
  e. Centrifuge the cell lysate at 24,878 × *g* for 1 h at 4°C.
  f. Filter the supernatants through 0.45 μm pore-sized Millex-HV Syringe Filter.

2. Affinity Chromatography based on His tag (Figure 2A)
  a. Wash a Ni^2+^-affinity column (HisTrap HP 5 mL) with 50 mL Histrap elution buffer A using a GE Healthcare AKTA prime plus FPLC System.
  b. Equilibrate the Ni^2+^-affinity column with 50 mL Histrap binding buffer A.
  c. Inject supernatants onto a Ni^2+^-affinity column at 3 mL/min at 4°C.
  d. Wash the column with 50 mL Histrap wash buffer A to remove impurities.
  e. Elute bound protein using a linear gradient of 4%–100% of Histrap elution buffer A.
  f. Analyze eluted proteins by 12% SDS-polyacrylamide gel electrophoresis (SDS-PAGE) and pool the fractions.

**Figure 2:**
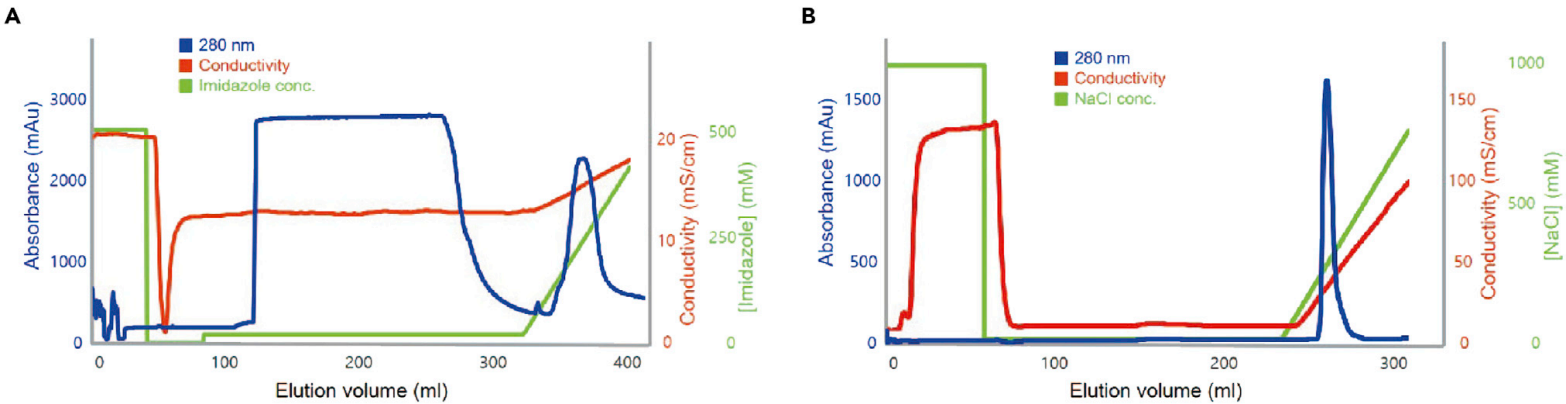
Elution profile after each step purification of LARS1 (A) Elution profile at 280 nm after Histrap chromatography of LARS1. (B) Elution profile at 280 nm after HiTrap Q FF chromatography of LARS1
3. Ion Chromatography (Figure 2B)
  a. Dilute eluted proteins with Q binding buffer A up to 10-fold.
  b. Wash an ion-exchange column (HiTrap Q FF) with 50 mL Q elution buffer A using a GE Healthcare AKTA prime plus FPLC system.
  c. Equilibrate the ion-exchange column with 50 mL Q binding buffer A.
  d. Inject the protein onto the ion-exchange column at 3 mL/min at 4°C.
  e. Wash the column with 100 mL Q binding buffer A.
  f. Elute the protein with a linear gradient of 0%–100% of Q elution buffer A.
  g. Analyze the eluted proteins by 12% SDS-PAGE and pool the fractions.
  h. Concentrate the protein to 2 mL with Amicon Ultra-15 Centrifugal Filter; 30 kDa cutoff.
4. Gel-filtration Chromatography
  a. Equilibrate a HiLoad 26/600 Superdex 200 pg gel-filtration column with 120 mL gel-filtration buffer at 1 mL/min using a GE AKTA Purifier 100 FPLC System.
  b. Centrifuge the protein preparation at 24,878 × *g* for 15 min at 4°C.
  c. Inject 2 mL of protein solution onto the gel-filtration column at 1 mL/min at 4°C.
  d. Flow with gel-filtration buffer at 1 mL/min at 4°C until the target protein is eluted.
  e. Analyze the purified protein by 12% SDS PAGE and pool the fractions.
  f. Concentrate the protein to ∼25 mg/mL with Amicon Ultra-15 Centrifugal Filter; 30 kDa cutoff.
  g. Store at −80°C.

**CRITICAL:** It is critical to maintain perform all the steps of protein purification at 4°C.

### Purification of LARS1 using reductive methylation

**Timing: 3 days**

In this step, we perform reductive methylation of LARS1 between the ion chromatography and the gel-filtration chromatography steps.

The affinity chromatography and ion chromatography were performed the same as in previous sections 1 to 3. Please use the HEPES buffer instead of Tris buffer for reductive methylation.

5. Reductive methylation
  a. Transfer 1 mL of protein at a concentration of 1 mg/mL to a microcentrifuge tube.
  b. Wrap the tube containing protein in aluminum foil.
  c. Add 20 μL of 1 M DMAB solution and 40 μL of 1 M formaldehyde solution to the protein at 4°C.
  d. Shake the tube at 4°C in the dark on a gel shaker maintained at 100 rpm for 2 h.
  e. Then, add again 20 μL of 1 M DMAB solution and 40 μL of 1 M formaldehyde solution to the protein at 4°C.
  f. Shake the tube at 4°C in the dark on a gel shaker maintained at 100 rpm for 2 h, again.
  g. Add 10 μL of 1 M DMAB solution and shake the tube at 4°C for 12–18 h.
  h. Add 125 μL of 1 M Tris-HCl pH 7.5 to quench the reaction.
  i. Subsequently, add 50 mM DTT to a final concentration of 1–5 mM to stabilize the protein.

**CRITICAL:** All operations should be performed with ice-cold reagents and samples, working either out of an ice bucket or in a cold room. Keep the samples tightly wrapped in aluminum foil between manipulations. HEPES or phosphate buffer are appropriate. The buffer should not contain amino groups and/or alcohols because such groups interfere with methylation of the sample. If necessary, additional salts (sodium chloride), polyols (glycerol), and other additives may be included to maintain protein stability, homogeneity, and solubility because they do not interfere with the reaction. This has proven successful with buffers of pH 6.0–8.0. The reaction is expected to proceed faster at higher pH values.

**Figure 3:**
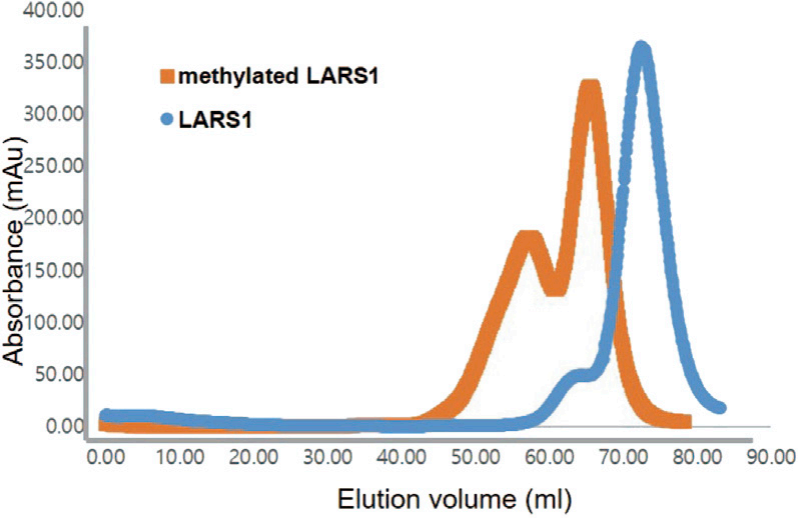
Elution profile at 280 nm after S200 gel filtration chromatography of LARS1 and methylated LARS1 The mass of methylated LARS1 (LARS1^methyl^) is increased compared to the mass of LARS1 because reductive methylation elevates the mass of the protein by 28 Da for each lysine residue present or could impact the conformation of LARS1 leading to a more extended conformer that elutes earlier then unmethylated LARS1.

6. Gel-filtration Chromatography (Figure 3)
  a. Equilibrate a HiLoad 26/600 Superdex 200 pg gel-filtration column with 120 mL gel- filtration buffer at 1 mL/min.
  b. Reduce the volume of the methylated protein to 2 mL with Amicon Ultra-15 Centrifugal Filter; 30 kDa cutoff.
  c. Centrifuge the protein at 24,878 × *g* for 15 min at 4°C.
  d. Inject the protein onto the gel-filtration column at 1 mL/min at 4°C.
  e. Flow with gel-filtration buffer at 1 mL/min at 4°C until the target protein has eluted.
  f. Analyze the purified protein by 12% SDS PAGE and pool the fractions.
  g. Concentrate the protein to ∼25 mg/mL with Amicon Ultra-15 Centrifugal Filter; 30 kDa cutoff.
  h. Store at −80°C.

### Measurement of binding affinity of leucine for LARS1 by microscale thermophoresis

**Timing: 1 day**

Microscale thermophoresis (MST) is a technology used for the biophysical analysis of interactions between molecules. It is used to monitor the movement of fluorescent molecules via a microscopic temperature gradient (Asmari et al., 2018). Overall, it is a frequently used method for quantitative characterization of intermolecular interactions and has many advantages such as low sample consumption, fast and cost-effective labeling (Bartoschik et al., 2018; Wienken et al., 2010).

In this step, we measure the binding affinity of leucine for LARS1 using a Monolith NT.115 instrument (Nano Temper Technologies) and then sought to co-crystalize LARS1 with leucine.

7. Purified LARS1 is diluted to a concentration of 400 nM in a volume of 100 μL with a buffer containing Tris-HCl pH 7.5, 150 mM NaCl, 2 mM DTT, 5 mM MgCl_2_, 0.1% Tween-20, and 0.05% BSA.
8. Mix 100 μL of LARS1 (400 nM) and 100 μL of RED-tris-NTA 2^nd^ Generation-dye (100 nM, Cat#MO-L018 in key resources table) to prepare Fluorescently-labeled LARS1.
9. Incubate for 30 min at 20–22°C.
10. Centrifuge the sample at 24,878 × *g* for 10 min at 4°C.

**CRITICAL:** Centrifuge the sample to avoid aggregation.

11. Prepare 25 μL of 10 mM leucine (final concentration 5 mM).
12. Add 10 μL of PBS buffer with 0.05% Tween-20 to PCR tubes 2–16.

**CRITICAL:** Prepare small microreaction tubes. Tubes with a volume of 200 μL or less were found to be suitable.

Thus, the effect of buffer dilution was avoided. The buffers in all tubes 1 to 16 were identical. Gradients of salt, DMSO, or other additives may interfere with the results.

13. Transfer 20 μL of leucine into PCR tube 1.
14. Transfer 10 μL of leucine from PCR tube 1 to PCR tube 2 and mix by pipetting up and down 3–4 times. Repeat for PCR tubes 3–16. Discard the extra 10 μL from PCR tube 16.

**CRITICAL:** Pipet up and down carefully to avoid bubbles or droplets.

**Figure 4:**
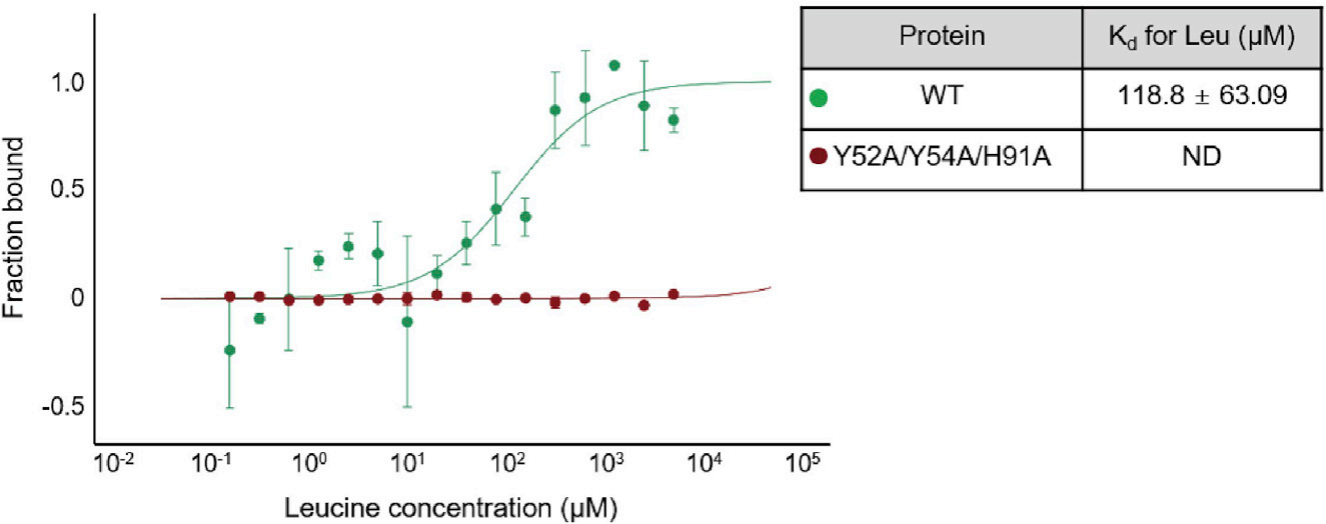
Binding affinity of leucine for LARS1 Binding affinity of leucine for LARS1 was determined using MST. The extract *K*_*d*_ values are listed in Figure 2 (n = 3; means ± SD). LARS1 WT binds to leucine, whereas LARS1 Y52A/Y54A/H91A does not bind to leucine. Figure reprinted with permission from Kim et al. (2021).

15. Add 10 μL of labeled LARS1 to each PCR tube (1–16) and mix by pipetting. The final LARS1 concentration is 100 nM. This concentration is used to calculate the *K*_*d*_ value.
16. Load the samples into capillaries by capillary action and place in a tray.
17. Measure the samples at 80% LED power using a green filter and 40% MST power.
18. The *K*_*d*_ is determined using the MO.Affinity Analysis *K*_*d*_ fit (Figure 4).

### Measurement inflection temperature (T_*i*_) of LARS1

**Timing: 1 h**

Differential Scanning Fluorimetry (DSF) measures protein unfolding by monitory changes in fluorescence as a function of temperature. DSF measures the Tryptophan fluorescence.

**Figure 5:**
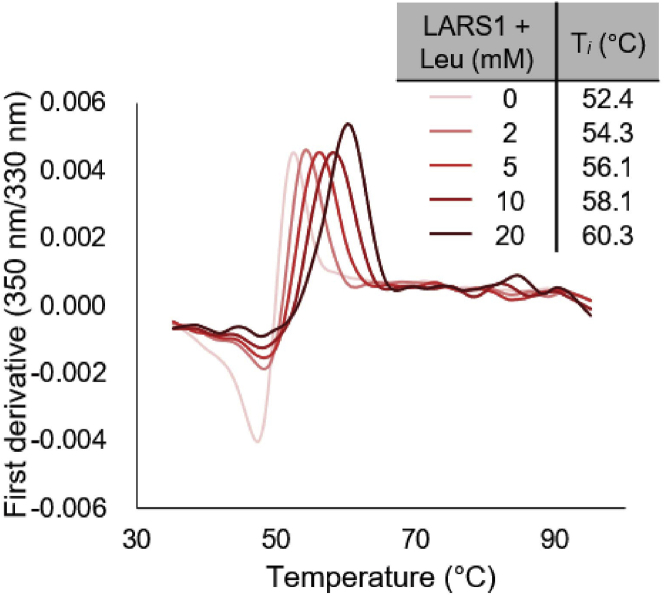
Inflection temperature (T_*i*_) of LARS1 with leucine The first derivatives of the fluorescence ratio (350–330 nm) are plotted. LARS1 was incubated with leucine and the inflection temperature (T_*i*_) was measured. The extracted T_*i*_ values are listed in Figure 3. Figure reprinted with permission from Kim et al. (2021).

19. Dilute the purified LARS1 to a concentration of 1 mg/mL with a buffer containing Tris-HCl pH 7.5, 150 mM NaCl, 2 mM DTT, 5 mM MgCl_2_, 0.1% Tween-20.
20. Transfer 20 μL LARS1 of into a PCR tube.
21. Add leucine to PCR tubes 2–5 at final concentrations of 2 mM, 5 mM, 10 mM, and 20 mM, respectively, and mix with LARS1.
22. Incubate 30 min at 4°C.
23. Load the samples into capillaries by capillary action and place in a tray.
24. Intrinsic fluorescence was recorded at 330 nm and 350 nm while heating the sample from 35 to 95°C at rate 3°C/min. The ratio of fluorescence (350/330 nm) and the T_*i*_ were calculated by Tycho NT. 6 (Figure 5).

### LARS1 post-crystallization soaking and cooling in cryoprotectants at −20°C and structure determination step

**Timing: 7–14 days**

In this step, we obtain crystals of LARS1-Leu^syn^, LARS1-ATP^syn^, LARS1-Leu-AMS^syn^, and LARS1^methyl^-Leu-AMS^syn^ (Kim et al., 2021). And then, we performed the post-crystallization soaking and cooling in cryoprotectants at −20°C (Rould et al., 1991; Heras and Martin, 2005). Finally, we collected the X-ray data and performed the structure determination by Phenix (Liebschner et al., 2019).

**Figure 6:**
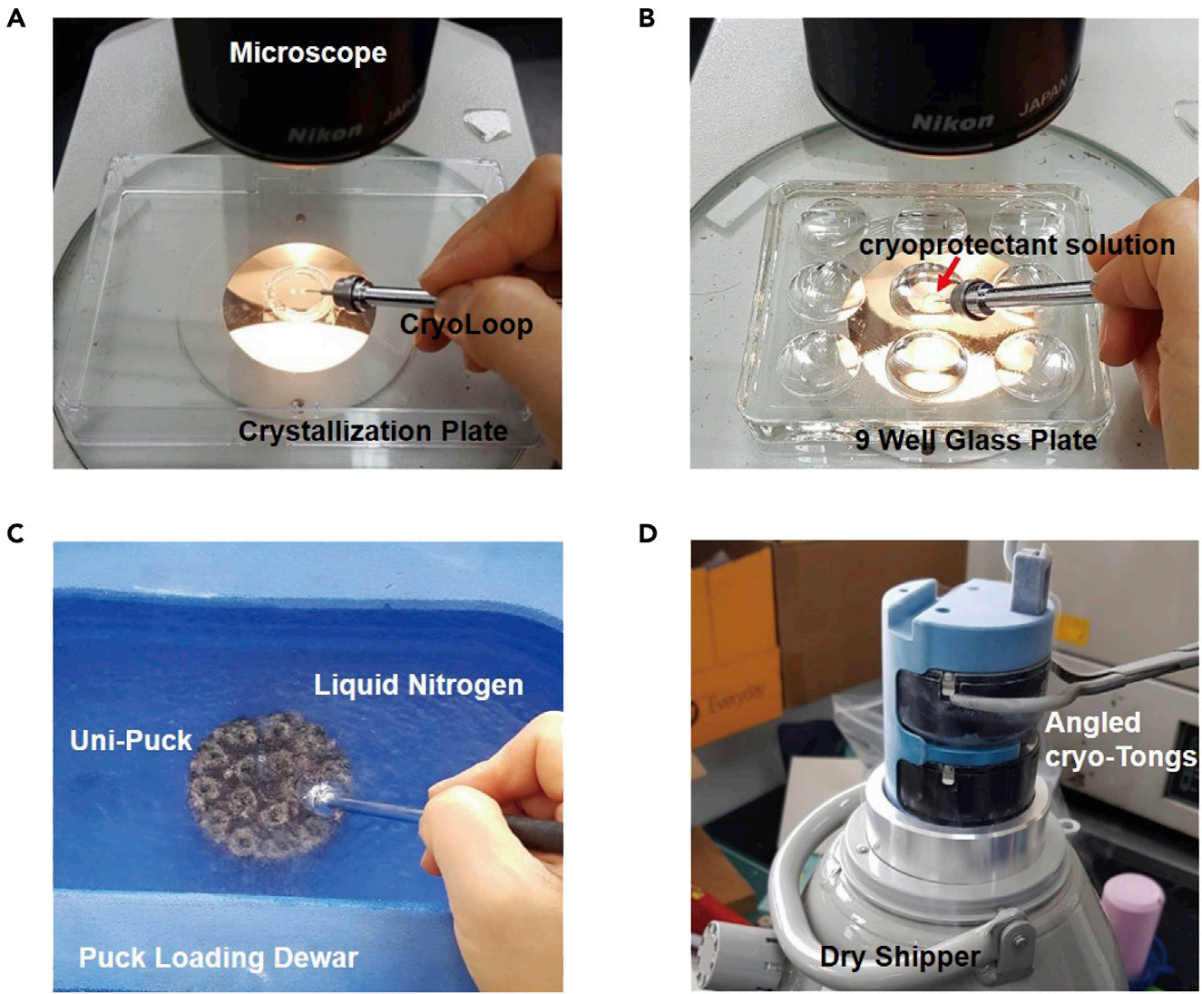
Post-Crystallization Soaking & Cooling in cryoprotectant of LARS1-Leu^syn^ crystals (A) Pick up the LARS1-Leu^syn^ crystals with a cryoloop. (B) Transfer the LARS1-Leu^syn^ crystals to 9 Well Glass Plate filled with 20 μL of cryoprotectant solution and seal with Crystal Clear sealing tape. Stabilize at −20°C for 1 day. (C) Transfer the LARS1-Leu^syn^ crystals to a Uni-Puck under liquid nitrogen. (D) Transfer the Uni-Puck to a dry shipper filled with liquid nitrogen until X-ray diffraction data collection.

25. Initial crystallization was performed at 20°C using the sitting-drop vapor diffusion method with an MRC 2-well crystallization plate and index kit (Hampton Research).
26. Thaw purified and frozen LAS1 and methylated LARS1 on ice.
27. Incubate LARS1 with different molecules (2 mM leucine for LARS1-Leu^syn^), (2 mM leucine and 1 mM ATP for LARS1-ATP^syn^), (2 mM leucine and 1 mM Leu-AMS for LARS1-Leu-AMS^syn^) and incubate the methylated LARS1 with (2 mM leucine and 1 mM Leu-AMS for LARS1^methyl^-Leu-AMS^syn^) for 1 h prior to crystallization.
28. LARS1-Leu^syn^ crystals are obtained by mixing 1 μL of ∼25 mg/mL LARS1 with 1 μL of a reservoir solution containing 0.1 M bis-tris pH 6.5, and 1.6 M ammonium sulfate at 20°C using the hanging-drop vapor diffusion method within 2–3 days. Crystals of LARS1-ATP^syn^, LARS1-Leu-AMS^syn^, and LARS1^methyl^-Leu-AMS^syn^ are obtained by mixing 1 μL of ∼17 mg/mL LARS1 or methylated LARS1 with 1 μL of reservoir solution containing 0.1 M HEPES pH 7.1, 0.42 M ammonium sulfate and 24% PEG 3350 at 20°C using the hanging-drop vapor diffusion method over 5 days.
29. Transfer the LARS1-Leu^syn^ crystals to a 9 Well Glass Plate filled with 20 μL of cryoprotectant solution containing 25% glycerol and 2 mM leucine in the reservoir using a cryoloop and seal with Crystal Clear sealing tape.
30. Immediately put the 9 Well Glass Plate in the −20°C freezer and then store for 1 day to stabilize.
31. Remove the plate with LARS1-Leu^syn^ crystals from the freezer.
32. Transfer LARS1-Leu^syn^ crystals to a Uni-Puck under liquid nitrogen using the cryoloop.
33. Transfer the Uni-Puck with LARS1-Leu^syn^ crystals to a dry shipper filled with liquid nitrogen and flash-freeze until X-ray diffraction data collection (Figure 6).

This method was used to prepare LARS1-Leu^syn^ crystals subsequently deposited in PDB with the code 6KQY.

**CRITICAL:** This step should be performed rapidly in a cold room (4°C). The crystals can be damaged at 20°C.

34. Transfer the crystals of LARS1-ATP^syn^, LARS1-Leu-AMS^syn^, and LARS1^methyl^-Leu-AMS^syn^ to a cryoprotectant solution containing 25% glycerol in reservoir solution and then immediately flash-freeze in liquid nitrogen.
35. Collect diffraction datasets at a synchrotron.
36. Index, integrate, and scale the images using HKL2000 (Otwinowski and Minor, 1997).
37. Obtain initial phases by molecular replacement (MR) using a search model (PDB entry: 1WKB) as an initial model, which was performed using Phaser (Murshudov et al., 1997). We found one molecule in asymmetric unit.
38. Density modification, including averaging and solvent flipping, are conducted using Solomon in CCP4i Suite, followed by automated model building based on density-modified data with the Autobuild module in PHENIX (Liebschner et al., 2019).
39. The all structures were refined with PHENIX (Liebschner et al., 2019).
40. The remaining residues are built manually using Coot (Emsley and Cowtan, 2004).

## Expected outcomes

Due to entropic effects, high concentrations of methylated lysine can yield a stabilized crystal through side chain interactions. The hydrophobic nature of methylated lysine is beneficial for interactions involving proteins and can change the interactions between proteins and solvents. These interactions can improve sample crystallization (Kim et al., 2008).

**Figure 7:**
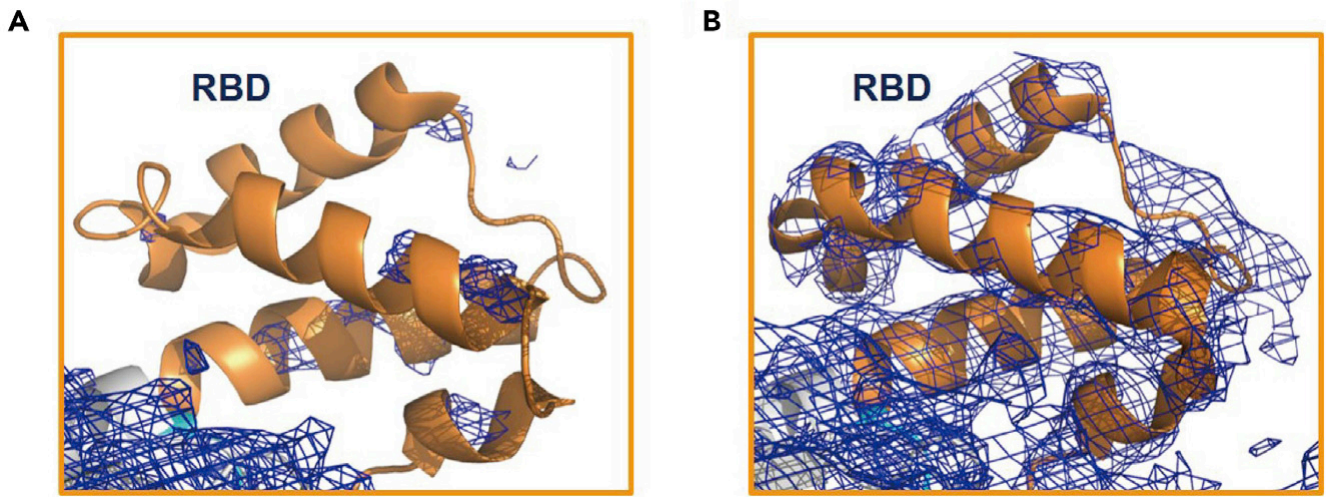
The ribbon model with electron density around RBD of LARS1 Comparison of electron density (2fo-fc map; 1.5 σ cutoff) of LARS1-Leu-AMS^syn^ (PDB entry: 6KIE) (A) and LARS1^methyl^-Leu-AMS^syn^ (PDB entry: 6KR7) (B) The RBD region (946-1015) is represented as an orange cartoon model. The resolution has greatly improved to ∼3.3 Å from ∼7.0 Å and the moscaicity (0.5 to 1.0) is much smaller than that of the original crystals (>1.5) (Figure 8).

Reductive methylated LARS1^methyl^-Leu-AMS^syn^ (PDB entry: 6KR7) showed improved electron density compared to LARS1-Leu-AMS^syn^ (PDB entry: 6KIE) (Figure 7).

**Figure 8:**
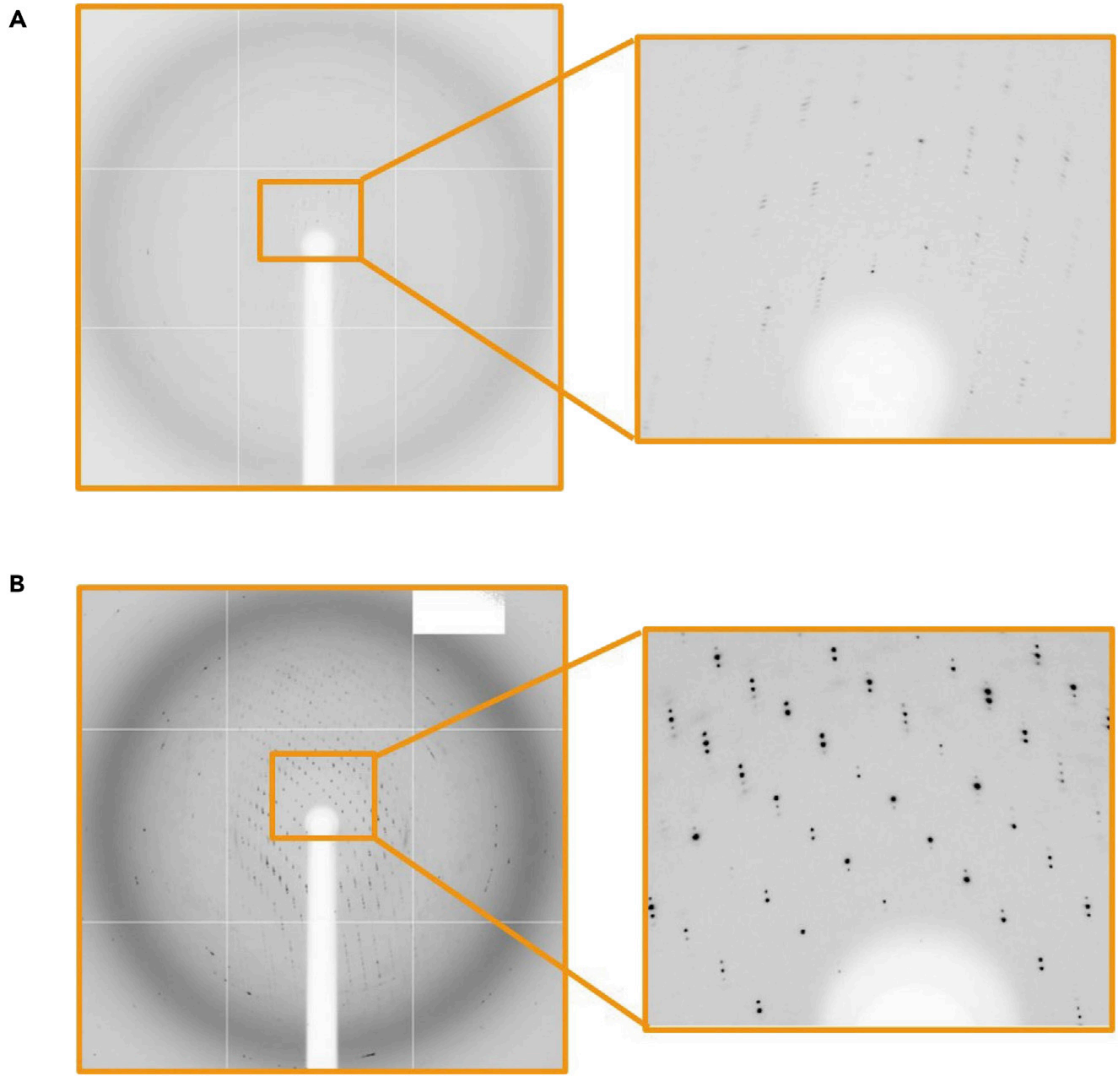
Photo image of X-ray diffraction Comparison of diffraction data for a crystal in cryoprotectant immediately (A) and that of a crystal stablized in cryoprotectant at −20°C for 1 day (B)

## Limitations

Not all lysine sites are reductively methylated. In some cases, the unmodified residue may be inaccessible to reagents and buried inside the protein or masked by bound ligands (Walter et al., 2006).

Not all crystals are stabilized by cryoprotectants. We sought to stabilize the crystals of LARS1-ATP^syn^, LARS1-Leu-AMS^syn^, and LARS1^methyl^-Leu-AMS^syn^ formed under the conditions of 0.1 M HEPES, pH 7.1, 0.42 M ammonium sulfate, and 24% PEG 3350, but the crystals melted or did not diffract.

## Troubleshooting

### Problem 1

While performing reductive methylation, the protein precipitates (step 5).

### Potential solution

Dilute the protein to a concentration of 1 mg/mL or less, because a higher concentration of protein leads to protein precipitation. If the amount of protein is high, the protein is transferred to a 15 mL or 50 mL centrifuge tube and 20 μL of 1 M DMAB solution and 40 μL of 1 M formaldehyde solution are added per mg/mL protein.

### Problem 2

**Figure 9:**
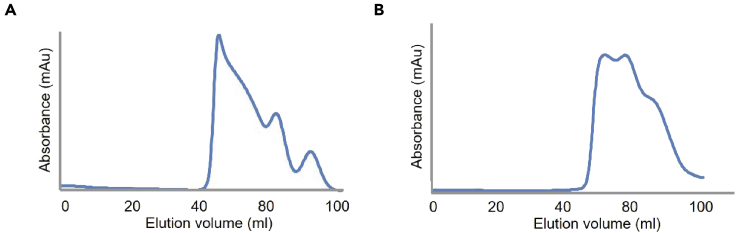
Elution data for LARS1 in ologomeric state with different buffer conditions (A) Elution data of LARS1 after reductive methylation using a buffer without Tween-20 (B) Elution data of LARS1 after reductive methylation using a buffer containing imidazole

When gel-filtration chromatography was performed after reductive methylation, the protein oligomeric state has changed (Figure 9) (step 6).

### Potential solution

Add additives to stabilize the protein. Methylation of LARS1 induced its oligomerization (Figure 9A). We added Tween-20 to the buffer, and reductive methylation of LARS1 was successfully performed.

The imidazole in the buffer was removed. When we performed reductive methylation after affinity chromatography, the oligomeric state of LARS1 protein has been changed because imidazole contains amino groups (Figure 9B). It is recommended to perform reductive methylation after buffer change or ion chromatography to remove imidazole.

### Problem 3

The buffer containing Tween-20 has a hazy precipitate (step 1).

### Potential solution

Prepare the buffer fresh the day before purification.

### Problem 4

**Figure 10:**
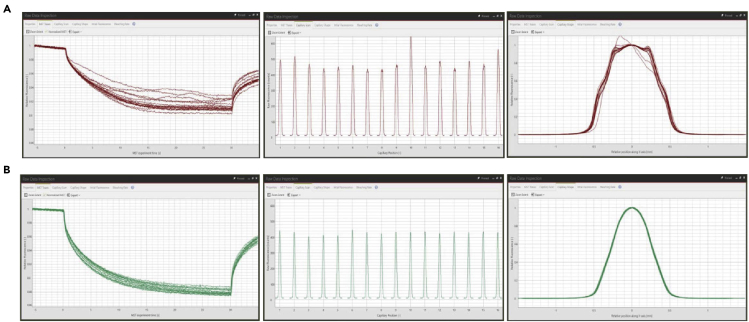
Proper analysis of MST raw data profiles with capillaries scan (A) Protein is absorbed to the inner surface of capillaries. Double fluorescence peaks appear. (B) Protein is not absorbed onto the inner surface of capillaries after treatment with 0.05% Tween-20 and 0.05% BSA in buffer A. Symmetrical fluorescence peaks are clearly shown for suitable analysis.

Double fluorescence peaks are observed. Some proteins are adsorbed to the inner surface of capillaries, resulting in an MST signal of poor quality (Figure 10A) (steps 17 and 18).

### Potential solution

Add detergent (e.g., 0.05% Tween-20, or 0.1% Pluronic F-127), and 0.05 % BSA to the buffer. In many cases, detergents improve sample homogeneity. Adjust the pH and salt concentration of the buffer to determine the optimal conditions and test different buffers. Check the concentration of organic solvents (e.g., DMSO). If adsorption continues, it is recommended to use premium quality capillaries (Figure 10B).

### Problem 5

**Figure 11:**
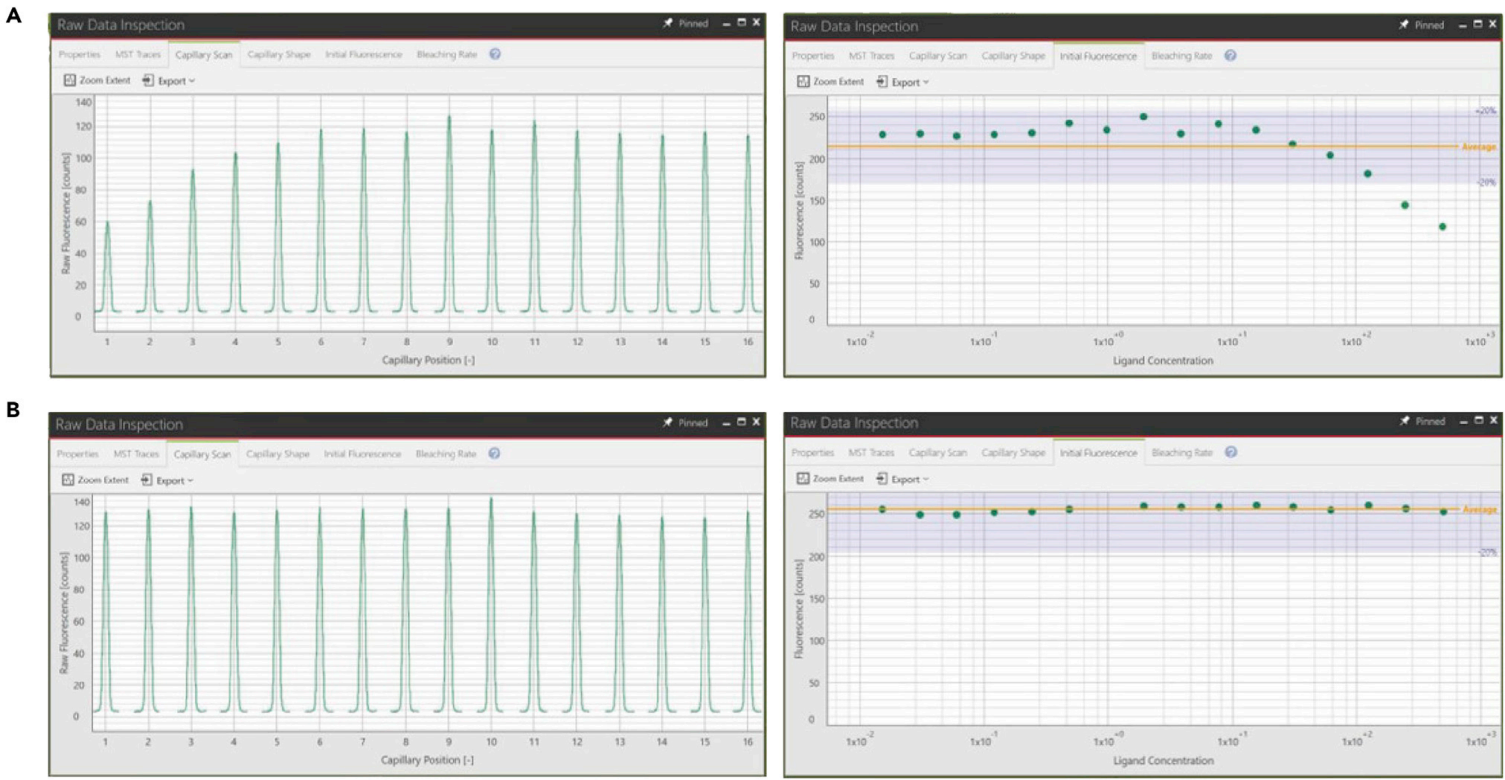
Proper analysis of MST raw data profiles with fluorescence quenching upon ligand binding (A) The fluorescence intensity decreases with increasing concentrations of the ligand. (B) The fluorescence is identical in all capillaries.

The fluorescence should be identical in all capillaries since the same amount of fluorescent molecule was added to each capillary. However, the fluorescence intensity often decreases with increasing concentrations of the ligand (Figure 11) (steps 17 and 18).

### Potential solution

Perform the SDS denaturation test (SD-test) that was developed for the analysis of the source of a ligand-induced fluorescence change that exceeds ±20% of the fluorescence average. The protocol is the denaturation of all proteins contained in the sample using a SD mix (4% SDS, 40 mM DTT) with heating to 95°C for 5 min. Load the denatured samples into capillaries and measure the fluorescence intensity. (1) If the fluorescence intensities for all samples are identical after the SD-test, it can be concluded that the previously observed fluorescence changes were induced by a binding event. Proceed with the next steps to determine the binding affinity directly from binding-related fluorescence changes. (2) If there is still a difference in fluorescence intensity after the SD-test, material was lost either by aggregation and subsequent centrifugation or by nonspecific adsorption to the plastics. Therefore, add detergent to the assay buffer (0.005% Tween-20, or 0.1% Pluronic F-127), in case the ligand-induced fluorescence change is caused by adsorption to the labware or aggregation of the target. Use non-binding reaction tubes or MTPs to avoid adsorption of biomolecules to lab-ware. In rare cases, the ligand might absorb the fluorescence of the target molecule even when it is not bound (inner filter effect). In this case, lowering the ligand concentration is recommended.

**CRITICAL:** It is essential to ensure that none of the pellet after centrifuging is transferred to the tubes with SD mix. If the pellet is disturbed, centrifuge again for at least 10 min ≥ 15,000 × *g*. The SD-test cannot be performed with samples containing potassium (200 mM or more) because the SDS will precipitate.

## Resource availability

### Lead contact

Further information and requests for resources and reagents should be directed to, and will be fulfilled by, the lead contact, Kwang Yean Hwang (chahong@korea.ac.kr).

### Materials availability

Unique and stable reagents generated in this study are available upon request.

### Data and code availability

The data supporting the findings of this study are available from Kim et al. (2021). The coordinates and structure factors for the crystal structures of human LARS1 have been deposited in the PDB under accession numbers 6KQY (LARS1-Leu^syn^), 6KID (LARS1-ATP^syn^), 6KIE (LARS1-Leu-AMS^syn^), and 6KR7 (LARS1^methyl^-Leu-AMS^syn^), respectively.

## References

[bib1] Adams P.D., Grosse-Kunstleve R.W., Hung L.-W., Ioerger T.R., McCoy A.J., Moriarty N.W., Read R.J., Sacchettini J.C., Sauter N.K., Terwilliger T.C. (2002). PHENIX: building new software for automated crystallographic structure determination. Acta Crystallogr. D Biol. Crystallogr..

[bib2] Asmari M., Ratih R., Alhazmi H.A., El Deeb S. (2018). Thermophoresis for characterizing biomolecular interaction. Methods.

[bib3] Bartoschik T., Galinec S., Kleusch C., Walkiewicz K., Breitsprecher D., Weigert S., Muller Y.A., You C., Piehler J., Vercruysse T. (2018). Near-native, site-specific and purification-free protein labeling for quantitative protein interaction analysis by MicroScale Thermophoresis. Sci. Rep..

[bib4] Emsley P., Cowtan K. (2004). Coot: model-building tools for molecular graphics. Acta Crystallogr. D Biol. Crystallogr..

[bib5] Han J.M., Jeong S.J., Park M.C., Kim G., Kwon N.H., Kim H.K., Ha S.H., Ryu S.H., Kim S. (2012). Leucyl-tRNA synthetase is an intracellular leucine sensor for the mTORC1-signaling pathway. Cell.

[bib6] Heras B., Martin J.L. (2005). Post-crystallization treatments for improving diffraction quality of protein crystals. Acta Crystallogr. D Biol. Crystallogr..

[bib7] Kim S., Yoon I., Son J., Park J., Kim K., Lee J.-H., Park S.-Y., Kang B.S., Han J.M., Hwang K.Y. (2021). Leucine-sensing mechanism of leucyl-tRNA synthetase 1 for mTORC1 activation. Cell Rep..

[bib8] Kim Y., Quartey P., Li H., Volkart L., Hatzos C., Chang C., Nocek B., Cuff M., Osipiuk J., Tan K. (2008). Large-scale evaluation of protein reductive methylation for improving protein crystallization. Nat. Methods.

[bib9] Kobayashi M., Kubota M., Matsuura Y. (1999). Crystallization and improvement of crystal quality for X-ray diffraction of maltooligosyl trehalose synthase by reductive methylation of lysine residues. Acta Crystallogr. D Biol. Crystallogr..

[bib10] Liebschner D., Afonine P.V., Baker M.L., Bunkõczi G., Chen V.B., Croll T.I., Hintze B., Hung L.-W., Jain S., McCoy A.J. (2019). Macromolecular structure determination using X-rays, neutrons and electrons: recent developments in Phenix. Acta Crystallogr. D Biol. Crystallogr..

[bib11] Murshudov G.N., Vagin A.A., Dodson E.J. (1997). Refinement of macromolecular structures by the maximum-likelihood method. Acta Crystallogr. D Biol. Crystallogr..

[bib12] Otwinowski Z., Minor W. (1997). [20] Processing of X-ray diffraction data collected in oscillation mode. Methods Enzymol..

[bib13] Rigsby R.E., Parker A.B. (2016). Using the P y MOL application to reinforce visual understanding of protein structure. Biochem. Mol. Biol. Educ..

[bib14] Rould M., Perona J., Steitz T. (1991). Structural basis of anticodon loop recognition by glutaminyl-tRNA synthetase. Nature.

[bib15] Walter T.S., Meier C., Assenberg R., Au K.-F., Ren J., Verma A., Nettleship J.E., Owens R.J., Stuart D.I., Grimes J.M. (2006). Lysine methylation as a routine rescue strategy for protein crystallization. Structure.

[bib16] Wienken C.J., Baaske P., Rothbauer U., Braun D., Duhr S. (2010). Protein-binding assays in biological liquids using microscale thermophoresis. Nat. Commun..

